# A consistency evaluation of signal-to-noise ratio in the quality assessment of human brain magnetic resonance images

**DOI:** 10.1186/s12880-018-0256-6

**Published:** 2018-05-16

**Authors:** Shaode Yu, Guangzhe Dai, Zhaoyang Wang, Leida Li, Xinhua Wei, Yaoqin Xie

**Affiliations:** 1 Shenzhen Institutes of Advanced Technology, Chinese Academy of Sciences, Shenzhen, China; 2 Shenzhen College of Advanced Technology, University of Chinese Academy of Sciences, Shenzhen, China; 3 Sino-Dutch Biomedical and Information Engineering School, Northeastern University, Shenyang, China; 4 School of Information and Control Engineering, Chinese University of Mining and Technology, Xuzhou, China; 5 Department of Radiology, Guangzhou First Peoples Hospital, Guangzhou Medical University, Guangzhou, China; 6 The Second Affiliated Hospital, South China University of Technology, Guangzhou, China

**Keywords:** Signal-to-noise ratio, Consistency evaluation, Medical image quality assessment, Magnetic resonance imaging

## Abstract

**Background:**

Quality assessment of medical images is highly related to the quality assurance, image interpretation and decision making. As to magnetic resonance (MR) images, signal-to-noise ratio (SNR) is routinely used as a quality indicator, while little knowledge is known of its consistency regarding different observers.

**Methods:**

In total, 192, 88, 76 and 55 brain images are acquired using T_2_^*^, T_1_, T_2_ and contrast-enhanced T_1_ (T_1_C) weighted MR imaging sequences, respectively. To each imaging protocol, the consistency of SNR measurement is verified between and within two observers, and white matter (WM) and cerebral spinal fluid (CSF) are alternately used as the tissue region of interest (TOI) for SNR measurement. The procedure is repeated on another day within 30 days. At first, overlapped voxels in TOIs are quantified with Dice index. Then, test-retest reliability is assessed in terms of intra-class correlation coefficient (ICC). After that, four models (BIQI, BLIINDS-II, BRISQUE and NIQE) primarily used for the quality assessment of natural images are borrowed to predict the quality of MR images. And in the end, the correlation between SNR values and predicted results is analyzed.

**Results:**

To the same TOI in each MR imaging sequence, less than 6% voxels are overlapped between manual delineations. In the quality estimation of MR images, statistical analysis indicates no significant difference between observers (Wilcoxon rank sum test, *p*_*w*_ ≥ 0.11; paired-sample *t* test, *p*_*p*_ ≥ 0.26), and good to very good intra- and inter-observer reliability are found (ICC, *p*_*icc*_ ≥ 0.74). Furthermore, Pearson correlation coefficient (*r*_*p*_) suggests that SNR_wm_ correlates strongly with BIQI, BLIINDS-II and BRISQUE in T_2_^*^ (*r*_*p*_ ≥ 0.78), BRISQUE and NIQE in T_1_ (*r*_*p*_ ≥ 0.77), BLIINDS-II in T_2_ (*r*_*p*_ ≥ 0.68) and BRISQUE and NIQE in T_1_C (*r*_*p*_ ≥ 0.62) weighted MR images, while SNR_csf_ correlates strongly with BLIINDS-II in T_2_^*^ (*r*_*p*_ ≥ 0.63) and in T_2_ (*r*_*p*_ ≥ 0.64) weighted MR images.

**Conclusions:**

The consistency of SNR measurement is validated regarding various observers and MR imaging protocols. When SNR measurement performs as the quality indicator of MR images, BRISQUE and BLIINDS-II can be conditionally used for the automated quality estimation of human brain MR images.

## Background

Medical image quality is highly related to many clinical applications, such as screening, abnormality detection and disease diagnosis. Nowadays, various kinds of imaging modalities are daily used, such as computerized tomography (CT) and magnetic resonance (MR) imaging, not to speak of these devices under development [1–3]. At the same time, massive medical images are collected and used to support the clinical decision making in each day. Therefore, how to evaluate the medical image quality wins increasing attention [4, 5].

Medical image quality assessment (MIQA) is crucial in the equipment quality assurance [6–8], comparison of algorithms for image restoration [9–13], image interpretation [14–17] and disease diagnosis [18, 19]. These MIQA algorithms can be grouped into the full- and no-reference categories [19–23]. The full-reference algorithms require the access to the reference image, while it is often unavailable in the medical imaging domain. To tackle this problem, the images from advanced devices are used as the reference to validate the proposed methods with images from common devices [24, 25]. However, this kind of approaches leads to new obstacles due to uncontrollable motion and particularly the different imaging characteristics. Comparatively, no-reference MIQA algorithms are more useful and challenging, and no reference information can be borrowed [20, 23, 26].

As a quality indicator of medical images, signal-to-noise ratio (SNR) is widely used to evaluate the development of new hardware and image processing algorithms [19, 23, 26–31]. The most common approach for SNR measurement, known as a “two-region” approach, is based on the signal statistics in two separate regions of interest (ROIs) from a single image. One is the tissue ROI (TOI) which determines the signal and the other ROI is localized in the object-free region which measures the noise [27, 28, 32]. The quality comparison of medical images with SNR measurement is still difficult across studies [23]. Above all, SNR values might vary according to the delineation of ROIs. For specific purposes, different tissues are concerned. And regarding the same purpose, it is impossible to delineate an identical tissue region. Moreover, the quality of MR imaging acquisition is closely related to the magnetic field strength (1.5 T, 3 T, etc), imaging protocol (T_1_, T_2_, etc), field of view (FOV), reconstruction methods and other significant factors. Furthermore, medical imaging is prone to unavoidable noise and artifacts. Besides, a great challenge might come from the fact that there are diverse imaging characteristics across modalities. Therefore, a consistency evaluation of SNR measurement is helpful in the further comparison of medical image quality.

In this paper, we evaluate the reliability of SNR measurement regarding different observers. At the preliminary stage, this study is confined to human brain MR images and four MR imaging sequences are analyzed. To the best of our knowledge, the most similar work is [26], in which it conducted the correlation analysis between subjective evaluation and 13 full-reference models. These models are primarily used for natural image quality assessment (NIQA). However, the study is with poor generalization. First, the experiment was based on synthesized distortions on 25 reference MR images and the result might be not so convincing in regard to real-life medical images. Second, the study involved subjective estimation to score the image quality, which is time consuming and expensive. On contrary, in this study, 411 in vivo human brain MR images are collected and 2 observers are involved to localize the tissue regions of white matter (WM) and cerebral spinal fluid (CSF) as the TOI for SNR measurement. Most importantly, this study investigates the SNR consistency regarding different observers. After the reliability of SNR measurement is verified, 4 no-reference NIQA models are borrowed from the computer vision community to predict the MR image quality, and furthermore, the correlation between the predicted results and SNR values is explored. On the whole, this study might shed some light on automated objective MIQA with less time and expenditure.

## Methods

### Data collection

In total, 192 T_2_^*^ weighted MR images of healthy brain, 88 T_1_, 76 T_2_ and 55 contrast enhanced T_1_ (T_1_C) weighted MR images of brain with cancerous tumors are collected. Participants were scanned with a 3.0 T scanner (Siemens, Erlangen, Germany) and an 8-channel brain phased-array coil was used.

Specifically, T_2_^*^ weighted images are acquired using gradient-echo pulse sequence. Its time of repetition (TR) is 200 ms and time of echo (TE) varies from 2.61 ms to 38.91 ms with an equal interval of 3.3 ms. The flip angle is 15^o^, FOV is 220 × 220 mm^2^, slice thickness is 3.0 mm and the resultant image matrix is 384 × 384. Note that the original purpose of multi-echo T_2_^*^ weighted image acquisition is toward tissue dissimilarity analysis [12]. T_1_, T_2_ and T_1_C weighted images are acquired using spin echo protocol with different TR and TE pairs (535 ms and 8 ms; 3500 ms and 105 ms; 650 ms and 9 ms). The flip angle is 15^o^, FOV is 220 × 220 mm^2^ and slice thickness is 1 mm or 2 mm. The resultant image size of T_1_ and T_1_C weighted MR images varies from 512 × 432 to 668 × 512, while the matrix size of T_2_ weighted MR images is ranged from 384 × 324 to 640 × 640.

### Image pre-processing

**Fig. 1:**
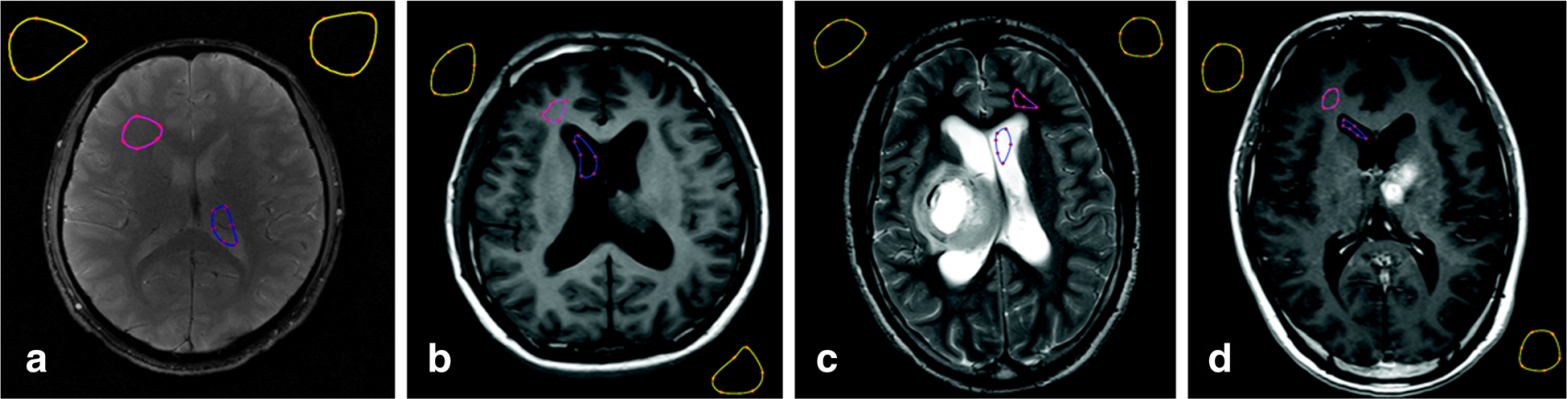
Manual outline of tissue regions and air regions. **a**, **b**, **c**, **d** are T_2_^*^, T_1_, T_2_ and T_1_C weighted MR images, respectively. **b**, **c**, **d** demonstrates one example of a subject. Primarily points localized by observers are noted with red sparkles. Outlined WM, CSF and AIR regions are in closed curves with pink, blue and yellow lines, respectively. Note that images have been cropped for display purpose

To each image, pixel intensity is linearly scaled to [0, 255]. Then, two TOIs (WM and CSF) are outlined in addition to two air regions. A non-physician (observer A, OA) and a radiologist with more than 15-year experience (observer B, OB) are asked to determine ROIs manually. Since the observers work separately and independently, they agree on that the size of outlined ROIs should be as large as possible. Furthermore, to T_1_, T_2_ and T_1_C weighted MR images, they also agree on that TOIs should be homogeneous and keep away from the tumor areas. The initial shape of each ROI is approximated with six points (the red sparkles in Fig. 1) and further refined by using a free-form curve-fitting method [33, 34]. The curve-fitting method takes the six points as the control points and Hermite cubic curve [35] is utilized for smooth interpolation between the points. In the end, outlined regions are as input to our in-house built algorithm with MATLAB (Mathworks, Natick, MA, USA) to measure the WM-based SNR (SNR_wm_) and CSF-based SNR (SNR_csf_) values. Note that the procedure is repeated on another day within 30 days for intra-observer reliability analysis.

Figure 1 shows T_2_^*^ (A), T_1_ (B), T_2_ (C) and T_1_C (D) weighted MR images. In each image, WM, CSF and AIR regions are in closed curves which are highlighted with pink, blue and yellow lines, respectively. Note that the red sparkles are primarily points localized by observers and images have been cropped for display purpose.

### SNR measurement

Two approaches exist for SNR measurement. The most common one requires two separate ROIs from a single image [27, 28]. By taking the signal (*S*) to be the average intensity in a tissue ROI (*μ*_*TOI*_) and the noise (*σ*) to be the standard deviation of the pixel intensity in a background ROI (*σ*_*AIR*_), we can approximate the SNR value of the image as below,

1 $$ {SNR}_{TOI}=\frac{S}{\sigma }=0.655\times \frac{\mu_{TOI}}{\sigma_{AIR}}. $$

Due to the Rician distribution of the background noise in a magnitude image, the factor of 0.655 arises because noise variations can be negative and positive [27, 28].

If the image is not homogeneous, the SNR measurement can be derived from the second approach [36, 37]. At first, a couple of images are acquired by consecutive scans and the MR device is equipped with identical imaging settings. And then, a difference image is derived by subtracting the images one from the other. Since the images are consecutively acquired on without any instability, the noise should be the only difference between the two original images. Taking the signal (*S*) as the mean pixel intensity value in a tissue ROI (*μ*_*oTOI*_) on one original image and the noise as the standard deviation (*σ*) in the same ROI on the subtracted image (*σ*_*sTOI*_),SNR can be estimated as

2 $$ {SNR}_{TOI}=\frac{S}{\sigma }=\sqrt{2}\times \frac{\mu_{oTOI}}{\sigma_{sTOI}}, $$

where the factor of $\sqrt{2}$ arises because the standard deviation (*σ*) is derived from the subtraction image but not from the original image.

This study utilizes Eq. (1) to measure SNR values of MR images, since image homogeneity is warranted in this study. In addition, the second approach is commonly used for equipment quality assurance and requires scanning the object twice.

### No-reference NIQA

Massive NIQA models are developed each year, while few models are used in the medical imaging community [38–40]. This study makes use of four automated no-reference NIQA methods to predict the MR image quality. The correlation analysis between SNR values and NIQA results aims to find potential no-reference NIQA models for MIQA applications.

Involved NIQA models utilize natural scene statistics (NSS) to estimate the general quality of natural images. Specifically, the blind image quality index (BIQI) [41] estimates the image quality based on the statistical features extracted in discrete wavelet transform (DWT). It requires no knowledge of the distortion types and can be extended to any kinds of distortions. The second indicator (BLIINDS-II) [42] is an improved version of blind image integrity notator using discrete cosine transform (DCT) statistics [38]. It adopts a general statistical model for score prediction. The third one, blind/referenceless image spatial quality evaluator (BRISQUE) [43], makes use of the locally normalized luminance coefficients and quantifies possible losses of “naturalness” which is a holistic measure of image quality. The last one is the natural image quality evaluator (NIQE) [44]. It builds a “quality-aware” selector that collects statistical features for natural image quality estimation.

These NIQA models are implemented with MATLAB (the Mathworks, Natick, MA, USA) and the codes provided by the authors are accessible online. The models are evaluated without modifications in this study. Full details of these algorithms can be referred to corresponding literature [41–44].

### Experiment design

The experiment is divided into three steps. First, the overlapping ratio of manually outlined TOIs between and within observers are concerned and Dice index is employed. The index is defined as $d=2\times \frac{\mid X\cap Y\mid }{\mid X\mid +\mid Y\mid}\times 100\%$, where X and Y stand for the TOI, and the signal ∣ ∣ indicates TOI computed as the number of voxels in the region. The Dice index equal to 100% means the two TOIs are identical, while it equal to 0% indicates the two TOIs are absolutely non-overlapping.

Then, with respect to the same TOI in each imaging sequence, the inter-observer difference is assessed with Wilcoxon rank sum test [45, 46] and paired-sample *t*-test [47]. The statistical analysis is performed using R (http://www.Rproject.org) and a significance level is set as 0.05. Moreover, the test-retest reliability is evaluated in terms of intra-class correlation coefficient (ICC, *p*_*icc*_) using a two-way mixed-effects model [48]. The values of *p*_*icc*_ ranging from 0.81 to 1.00 suggest very good reliability and 0.61 to 0.80 good reliability.

In the end, the correlation between SNR values and NIQA results is analyzed by using Pearson correlation coefficient (*r*_*p*_) [49]. Note that the values of *r*_*p*_ ranging from 0.81 to 1.00 indicate very strong or good correlation, while 0.61 to 0.80 good or strong correlation.

## Results

### Overlapped voxels in TOIs

Table 1 summarizes the number of voxels in TOIs in each MR sequence (the mean and standard deviation, *μ* ± *σ*). It is found that hundreds of voxels are outlined for SNR measurement and the minimum is 330±72.

**Table 1:** The number of voxels in the outlined tissue regions

		T_2_^*^		T_1_		T_2_		T_1_C	
		WM	CSF	WM	CSF	WM	CSF	WM	CSF
The first time	OA	423 (95)	381 (117)	558 (173)	614 (258)	609 (239)	889 (366)	523 (146)	704 (314)
	OB	**330** (72)	333 (138)	567 (181)	649 (318)	414 (174)	699 (288)	477 (156)	663 (272)
The second time	OA	382 (88)	378 (104)	530 (187)	626 (219)	589 (251)	853 (349)	505 (138)	692 (290)
	OB	357 (119)	342 (119)	582 (176)	663 (282)	447 (195)	721 (306)	480 (177)	686 (268)

Specifically, the overlapping ratio is described with Dice index as shown in Table 2. It indicates that less than 6% voxels are overlapped between and within observers in the manual delineation of TOIs.

**Table 2:** Dice index for the overlapped percentage of voxels in the TOIs between and within observers

		WM		CSF		
		OB1	OB2	OA2	OB1	OB2
T_2_^*^	OA1	0.05	0.03	0.05	0.04	0.03
	OA2	0.03	0.04		0.03	0.03
	OB1		0.06			0.06
T_1_	OA1	0.02	0.03	0.03	0.04	0.03
	OA2	0.03	0.03		0.01	0.02
	OB1		0.02			0.02
T_2_	OA1	0.02	0.04	0.02	0.02	0.01
	OA2	0.03	0.03		0.03	0.02
	OB1		0.02			0.02
T_1_C	OA1	0.02	0.02	0.03	0.02	0.03
	OA2	0.02	0.03		0.01	0.03
	OB1		0.04			0.02

### Analysis of SNR values

**Fig. 2:**
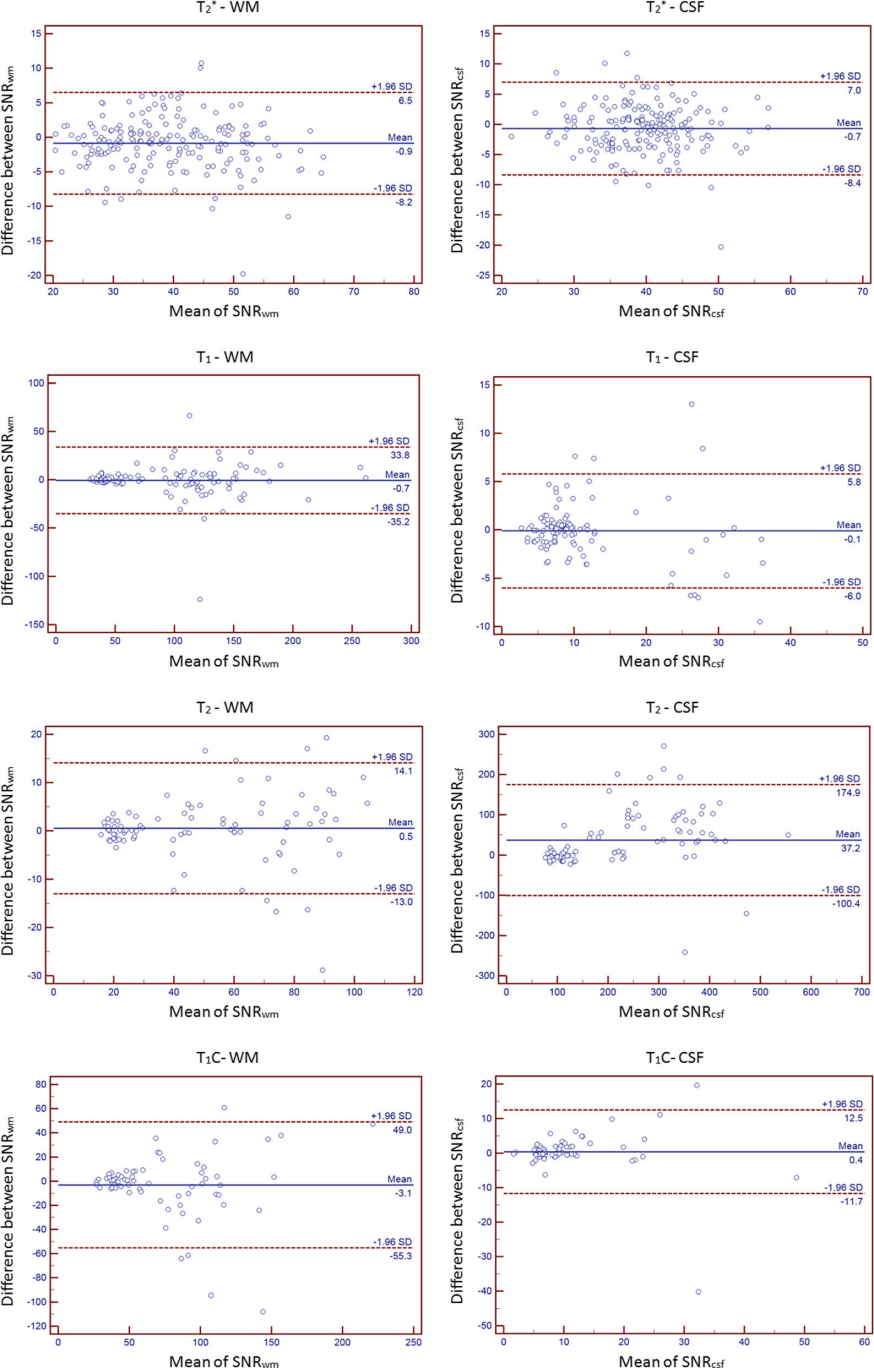
Bland & Altman plots of SNR values. It presents the SNR values of the first time measurement. The left column represents SNR_wm_ values and the right shows SNR_csf_ values. The solid lines indicate the mean values of SNR measurements and the dashed lines indicate the 95% confident interval of the difference between observations

Figure 2 shows the first-time measurement of SNR values by using Bland & Altman plots [50]. It is a scatter diagram of the differences plotted against the averages of two SNR observations. In each plot, the average and the difference of SNR values can be perceived from the horizontal and the vertical axis respectively. In addition, horizontal lines are drawn at the mean difference between two SNR observations and at the limits of agreement. The latter is defined as the mean difference plus and minus 1.96 times the standard deviation (SD) of the SNR difference. The Bland & Altman plots show that more than 89% points are localized between the limits of agreement.

### Inter-observer difference

Inter-observer difference of SNR observations is analyzed with Wilcoxon rank sum test (*p*_*w*_) and paired-sample *t* test (*p*_*p*_). Corresponding results are show in Table 3. Note that the minimum value is boldfaced in each test. It is observed that the minimal *p*_*w*_ is 0.11 and *p*_*p*_ is 0.26. It is also found that both *p*_*w*_ and *p*_*p*_ from SNR_wm_ are larger than those from SNR_csf_, correspondingly.

**Table 3:** Statistical analysis of SNR measure in each imaging sequence regarding different TOIs

		T2*		T1		T2		T1C	
		WM	CSF	WM	CSF	WM	CSF	WM	CSF
The first time	*p*w	0.54	0.39	0.88	0.74	0.99	**0.11**	0.69	0.56
	*p*p	0.41	0.30	0.98	0.59	0.94	**0.28**	0.77	0.46
The second time	*p*w	0.57	0.33	0.92	0.75	0.95	**0.18**	0.72	0.58
	*p*p	0.44	0.36	0.96	0.62	0.96	**0.26**	0.79	0.47

### Test-retest reliability

Table 4 lists the result of test-retest reliability. Note that ICC_1_ and ICC_2_ respectively stands for intra- and inter-observer correlation coefficient. As shown in the Table, very good intra-observer reliability of the experience radiologist (OB) is found (*p*_*icc*_ ≥ 0.81). Similar results are found on the non-physician (OA) except that only good reliability is achieved for SNR_csf_ on T_2_^*^ (*p*_*icc*_ ≥ 0.79) and T_2_ (*p*_*icc*_ ≥ 0.76) weighted MR images. Furthermore, good to very good inter-observer reliability is found (*p*_*icc*_ ≥ 0.80) but only good inter-observer reliability is found for SNR_csf_ in T_2_^*^ weighted MR imaging sequence (*p*_*icc*_ ≥ 0.74).

**Table 4:** Intra- and inter-observer reliability in terms of intra-class coefficients between the non- and experienced physician

		T2*		T1		T2		T1C	
		WM	CSF	WM	CSF	WM	CSF	WM	CSF
Intra-observer reliability	OA	0.84	0.79	0.91	0.87	0.95	0.76	0.89	0.86
	OB	0.86	0.81	0.95	0.83	0.97	0.85	0.88	0.82
Inter-observer reliability	ICC2	0.81	0.74	0.92	0.80	0.90	0.81	0.85	0.83

### Correlation between SNR and NIQA

Table 5 shows the correlation coefficients (*r*_*p*_) between mean SNR values of each TOI (two measurements each observer) and NIQA results. The bold-faced *r*_*p*_ values in red and blue denote *r*_*p*_ ≥ 0.60. Specifically, to SNR_wm_, BIQI, BLIINDS-II and BRISQUE on T_2_^*^ (*r*_*p*_ ≥ 0.78), BRISQUE and NIQE on T_1_ (*r*_*p*_ ≥ 0.77), BLIINDS-II on T_2_ (*r*_*p*_ ≥ 0.68), and BRISQUE and NIQE on T_1_C (*r*_*p*_ ≥ 0.62) images show strong correlation; while to SNR_csf_ values, BLIINDS-II correlates well on T_2_^*^ (*r*_*p*_ ≥ 0.63) and T_2_ (*r*_*p*_ ≥ 0.64) weighted MR imaging sequence.

**Table 5:** Correlation between TOI-based SNR values and no-reference NIQA results

	T2*				T1				T2				T1C			
	SNRwm		SNRcsf		SNRwm		SNRcsf		SNRwm		SNRcsf		SNRwm		SNRcsf	
	OA	OB	OA	OB	OA	OB	OA	OB	OA	OB	OA	OB	OA	OB	OA	OB
BIQI	**0.81**	**0.79**	0.55	0.57	0.16	0.11	0.15	0.13	0.18	0.25	0.07	0.29	0.36	0.33	0.08	0.12
BLIINDS-II	**0.78**	**0.80**	**0.72**	**0.63**	0.23	0.20	0.02	0.06	**0.72**	**0.68**	**0.73**	**0.64**	0.34	0.38	0.10	0.15
BRISQUE	**0.82**	**0.81**	0.56	0.52	**0.77**	**0.81**	0.18	0.22	0.45	0.37	0.52	0.28	**0.62**	**0.73**	0.33	0.36
NIQE	0.24	0.27	0.35	0.03	**0.82**	**0.84**	0.24	0.28	0.55	0.46	0.53	0.32	**0.63**	**0.72**	0.32	0.30

## Discussion

This paper has validated the consistency of SNR measurement in the quality assessment of human brain MR images. Moreover, the correlation between TOI-based SNR measurement and NIQA models has been analyzed. The study suggests that off-the-shelf NIQA models used in computer vision community are full of potential for automated and objective MIQA applications.

The consistency evaluation indicates that SNR measurement is reliable to different observers in each MR imaging sequence. In image pre-processing, TOIs are randomly localized. When no overlapping between TOIs, the Dice index would be zero. On average, TOIs are slightly overlapped by no more than 6% [Table 2], while the statistical analysis indicates that SNR values are not significantly changed between observers [Table 3]. That means independent localization of TOIs makes no difference to SNR measurement. Moreover, the test-retest reliability study suggests good to very good intra- and inter-observer reliability (Table 4). That might be the reason why SNR is widely used in clinical situations. And accordingly, a non-physician can independently perform the SNR measurement of MR images as good as an experienced physician does.

The correlation between SNR values and NIQA models shows that BLIINDS-II correlates well with SNR_csf_ on T_2_^*^ and T_2_ weighted MR images, since CSF presents relatively higher voxel intensity over other tissues that leads to the robust estimation of SNR_csf_. In comparison to SNR_csf_, more NIQA results are in good correlation with SNR_wm_ values, since WM is distinguishable in involved MR imaging sequences. Therefore, the authors suggest that tissue regions with higher intensities should function as the TOI in SNR measurement. On the whole, BRISQUE performs well as an automated no-reference NIQA model for the quality assessment of T_2_^*^, T_1_ and T_1_C weighted MR brain images, and BLIINDS-II is superior on assessing the quality of T_2_^*^ and T_2_ MR images independent of the TOI selection. Consequently, it is full of potential to modify NIQA models developed in the computer vision community for MIQA applications in the medical imaging domain [51]. It should be mentioned that the correlation of SNR values and predicted results is not very good (*r*_*p*_ ≤ 0.85) and further improvement or modifications of existing NIQA models is needed.

SNR is frequently used as an image quality indicator in clinic. It is a local measure regarding the whole MR image. The SNR measurement can also be formulated from the global signal by using the whole object region as the tissue region. An overview of existing definitions of SNR measurement can be referred to [23]. More general and automated MIQA algorithms include using Shannon’s theory to describe the image content and then to model the spatial spectral power density of the image as the quality indicator [21] or analyzing the background of magnitude images of structural brain to represent the image quality [52]. In particular, some researchers explore to bridge the gap between SNR measurement and diagnostic accuracy or detectability [9, 18]. These studies show superiority over the physical measure of image quality, since the ultimate goal of medical imaging aims at abnormality detection and disease diagnosis.

## Conclusions

The consistency of SNR measurement is validated regarding different observers. The correlation between SNR measurement and NIQA models indicates that BRISQUE works well for automated MIQA of T_2_^*^, T_1_ and T_1_C weighted brain MR images, and BLIINDS-II is superior over T_2_^*^ and T_2_ weighted images independent of the TOI selection. Our future work will focus on the connection of SNR measurement, NIQA models and MIQA applications.

## Abbreviations

BIQI: Blind image quality index
BLIINDS-II: The improved version of blind image integrity notator using DCT statistics
BRISQUE: Blind/referenceless image spatial quality evaluator
CSF: Cerebral spinal fluid
CT: Computerized tomography
DCT: Discrete cosine transform
DWT: Discrete wavelet transform
FOV: Field of view
ICC_1_: Intra-observer correlation coefficient
ICC_2_: Inter-observer correlation coefficient
MIQA: Medical image quality assessment
MR: Magnetic resonance
NIQA: Natural image quality assessment
NIQE: Natural image quality evaluator
NSS: Natural scene statistics
OA: Observer A
OB: Observer B
ROI: Regions of interest
SD: Standard deviation
SNR: Signal-to-noise ratio
SNR_csf_: CSF-based SNR
SNR_wm_: WM-based SNR
T_1_C: Contrast-enhanced T_1_
TE: Time of echo
TOI: Tissue region of interest
TR: Time of repetition
WM: White matter
